# A urinalysis to urine culture reflex protocol results in high rates of asymptomatic bacteriuria treatment

**DOI:** 10.1017/ash.2025.10130

**Published:** 2025-09-12

**Authors:** Dina Zheng, Lina Hamid, Morgan L. Bixby, Elizabeth B. Hirsch

**Affiliations:** 1 University of Minnesota College of Pharmacy, Minneapolis, MN, USA; 2 M Health Fairview University of Minnesota Medical Center, Minneapolis, MN, USA

## Introduction

Asymptomatic bacteriuria (ASB) is defined as the presence of bacteria in the urine without signs or symptoms of urinary tract infection (UTI).^1^ Despite IDSA guideline recommendations against ASB treatment in most adults, between 45% and 83% of patients receive inappropriate antimicrobial treatment, often due to an emphasis on laboratory results rather than symptoms.^2–6^ At our institution, a urinalysis (UA) positive for ≥1 criteria (pyuria [>10 white blood cells/high-power field (WBC/HPF)], “moderate” or “large” leukocyte esterase, or nitrites) results in a reflex to urine culture (UC). The objective of this study was to assess the frequency and specific criteria driving UC reflex orders, incidence of ASB, and ASB treatment rate to identify associations between urine diagnostics and UTI symptoms.

## Methods

Retrospective data for patients ≥18 years old and hospitalized at an 850-bed academic medical center, from November 2022 to February 2023, were extracted from the electronic health record using the Best Practices Integrated Informatics Core. Data included baseline demographics, microbiological results, clinical data, and antibiotic use. Patients with a UA order and UC within 72 hours of UA were screened for inclusion. Patients were excluded if they were pregnant or undergoing an invasive urologic procedure. The number and frequency of UC reflex and UA criteria driving reflex were assessed. RStudio (v.2023.06.0) was utilized to perform logistic regression analyses with univariate and multivariate models to calculate odds ratios for UC reflex.

Additional retrospective chart review using REDCap® was completed for patients with a UA/UC collected in the first and last month of the data pull. Immunocompromised patients including solid organ transplant (*n* = 7) and bone marrow transplant (*n* = 2) patients were included. Only patients with reports of lower UTI symptoms were reviewed for the presence of upper UTI symptoms. Only the index UA order was included for analysis if ≥1 UA/UC order was collected per encounter. Encounters with antibiotics started prior to UA/UC were excluded. Empiric therapy was defined as antibiotics started for UTI prior to the UC result date or if the UC was negative, and definitive therapy was defined as antibiotics started based on UC results. The University of Minnesota institutional review board determined this study met criteria for exemption from review (IRB ID: STUDY00020351).

## Results

This study comprised 2,226 unique patients. Median age was 61 years (IQR 45–71) and 50.4% were female. Of 3,072 total UA orders, 926 (30.1%) reflexed to UC. The highest frequency of UC reflex occurred with 2/3 UA criteria positive (51.1%), followed by 1/3 criteria (36.7%), and then 3/3 criteria (12.2%). In both the univariate and multivariate models, pyuria had the highest odds of triggering UC reflex (Table 1).

**Table 1.** Logistic regression analyses for urinalysis (UA) criteria trigger for urine culture (UC) reflex

UA criteria	Univariate model		Multivariate model	
	OR (95% CI)	*p* value	OR (95% CI)	*p* value
Pyuria (>10 WBC/HPF)	2.61 (2.57–2.64)	<.001	2.23 (2.20–2.27)	<.001
Leukocyte esterase	2.40 (2.34–2.46)	<.001	1.20 (1.18–1.23)	<.001
Nitrites	2.10 (1.96–2.24)	<.001	1.28 (1.25–1.31)	<.001

OR, odds ratio; CI, confidence interval; >10 WBC/HPF, >10 white blood cells/high-power field.

In the retrospective chart review cohort, 128 of 292 (43.8%) of UC reflex orders were positive with the majority (69.5%) in patients with ASB, defined as absence of any lower/upper UTI symptoms (Supplemental Table 1). Overall, UTI symptoms were present in 28.4% of encounters with dysuria being the most common (15.1%). Upper UTI symptoms were present in 21.9% of encounters, most frequently nausea/vomiting (13.0%). Systemic signs of infection were identified in 61.3% of encounters with tachycardia (54.8%) most frequent (Supplemental Table 2). Altered mental status changes were present in 24.3% of encounters among patients with a median age of 68 years.

Over half (65.1%) of patient encounters had antibiotics started for UTI, with 95.3% started based on timing after UA results (ie, prior to UC results), and 80.9% of ASB encounters were treated with antibiotics. A mean 1.9 antibiotics (SD 1.1) were started per encounter, with third-generation cephalosporins being the most common for both empiric and definitive therapy (Supplemental Figures 1–3). Median total length of therapy was 6 days (IQR 3–10), with 3 days (IQR 3–6) for empiric versus 7 days (IQR 4.5–10.5) for definitive therapy.

Concurrent non-UTI infection(s) were present in 46.2% of encounters. Sub-analysis (*n* = 157) findings after excluding these encounters identified similar rates of antibiotic treatment (68.8% vs 65.1%) and antibiotics started based on UA results (91.7% vs 95.3%) compared to the entire cohort. This subgroup also demonstrated a higher rate of bacteriuria (60.0% vs 43.8%) but similar rate of ASB (72.5% vs 69.5%) and ASB treatment (79.3% vs 80.9%).

## Discussion

Despite frequent UA orders, only 30.1% reflexed to UC. Most UC reflexes were due to one or two UA criteria positive with pyuria having the highest odds of triggering UC reflex. Of the UC reflex orders, over 50% yielded negative results, contributing to an unnecessarily high laboratory workload. There was a high frequency of antibiotic treatment initiated based on UA results and/or for ASB.

This study has several limitations. Almost half of patients had a concurrent non-UTI infection and broad infection workup, hence empiric antibiotics were often started to cover multiple potential infection sources. A sub-analysis was completed to address this potential confounding factor, with findings remaining consistent after excluding patients with other concomitant infections. Patients with certain conditions (eg, neurogenic bladder, kidney transplant) may have atypical UTI presentations, making it difficult to differentiate symptomatic UTI from ASB.

Our findings demonstrate high volumes of urine diagnostic test orders with predominantly negative results. Additionally, most urine diagnostic orders were placed for asymptomatic patients, signifying a high burden of unnecessary laboratory time and resources. These results highlight the need for diagnostic stewardship measures to address inappropriate UA/UC orders and ASB treatment. Diagnostic stewardship interventions may include a combination of clinical decision support tools and provider education, which have demonstrated effectiveness in decreasing UA/UC orders and antibiotic use.^7–10^

## Supporting information

10.1017/ash.2025.10130.sm001 Zheng et al. supplementary material Zheng et al. supplementary material

## Data Availability

The data are not openly available due to ethical considerations.

## Supplementary material

The supplementary material for this article can be found at https://doi.org/10.1017/ash.2025.10130.
