# Supplementary material for: A urinalysis to urine culture reflex protocol results in high rates of asymptomatic bacteriuria treatment
Source: Antimicrob Steward Healthc Epidemiol. 2025 Sep 12;5(1):e213. doi: 10.1017/ash.2025.10130 (PMC12451803; doi:10.1017/ash.2025.10130)
Supplement: Zheng et al. supplementary material [file S2732494X25101307sup001.docx]

**Supplemental Table 1.** Type and frequency of urinary tract infection (UTI) symptoms among patients with a urine culture (UC) reflex.

| UTI Symptoms | Patients with UC Reflex *N* = 292 |
| --- | --- |
| Any lower UTI symptom^a^, *n* (%) | 83 (28.4) |
| Dysuria | 44 (15.1) |
| Urinary frequency | 31 (10.6) |
| Acute hematuria | 25 (8.6) |
| Suprapubic pain/tenderness | 23 (7.9) |
| Urinary urgency | 17 (5.8) |
| Any upper UTI symptom^b^, *n* (%) | 64 (21.9) |
| Nausea/vomiting | 38 (13.0) |
| Fever/chills | 33 (11.3) |
| CVA pain/tenderness | 31 (10.6) |
| Malaise | 26 (8.9) |

CVA, costovertebral angle

^a^Lower UTI symptoms were defined as any of the below urinary symptoms in the absence of systemic symptoms.

^b^Upper UTI symptoms were defined as any of the below urinary symptoms in the presence of systemic symptoms. Patients were only reviewed for presence of upper UTI symptoms if they had reports of lower UTI symptoms.

**Supplemental Table 2.** Type and frequency of systemic symptoms among patients with a urine culture (UC) reflex.

| Systemic Signs | Patients with UC Reflex *N* = 292 |
| --- | --- |
| Any systemic signs, *n* (%) | 179 (61.3) |
| HR >90 bpm | 160 (54.8) |
| WBC <4000 or >12,000 cells/mm^3^ | 118 (40.4) |
| RR >20 breaths/min | 115 (39.4) |
| Temperature <36 ºC or >38 ºC | 71 (24.3) |
| SBP <90 mmHg | 53 (18.2) |

HR, heart rate; bpm, beats per minute; WBC, white blood cell; RR, respiratory rate; SBP, systolic blood pressure

**Supplemental Figure 1.** Number of antibiotics started for urinary tract infection (UTI) per patient encounter among patients (n = 292) with a urine culture (UC) reflex.

**
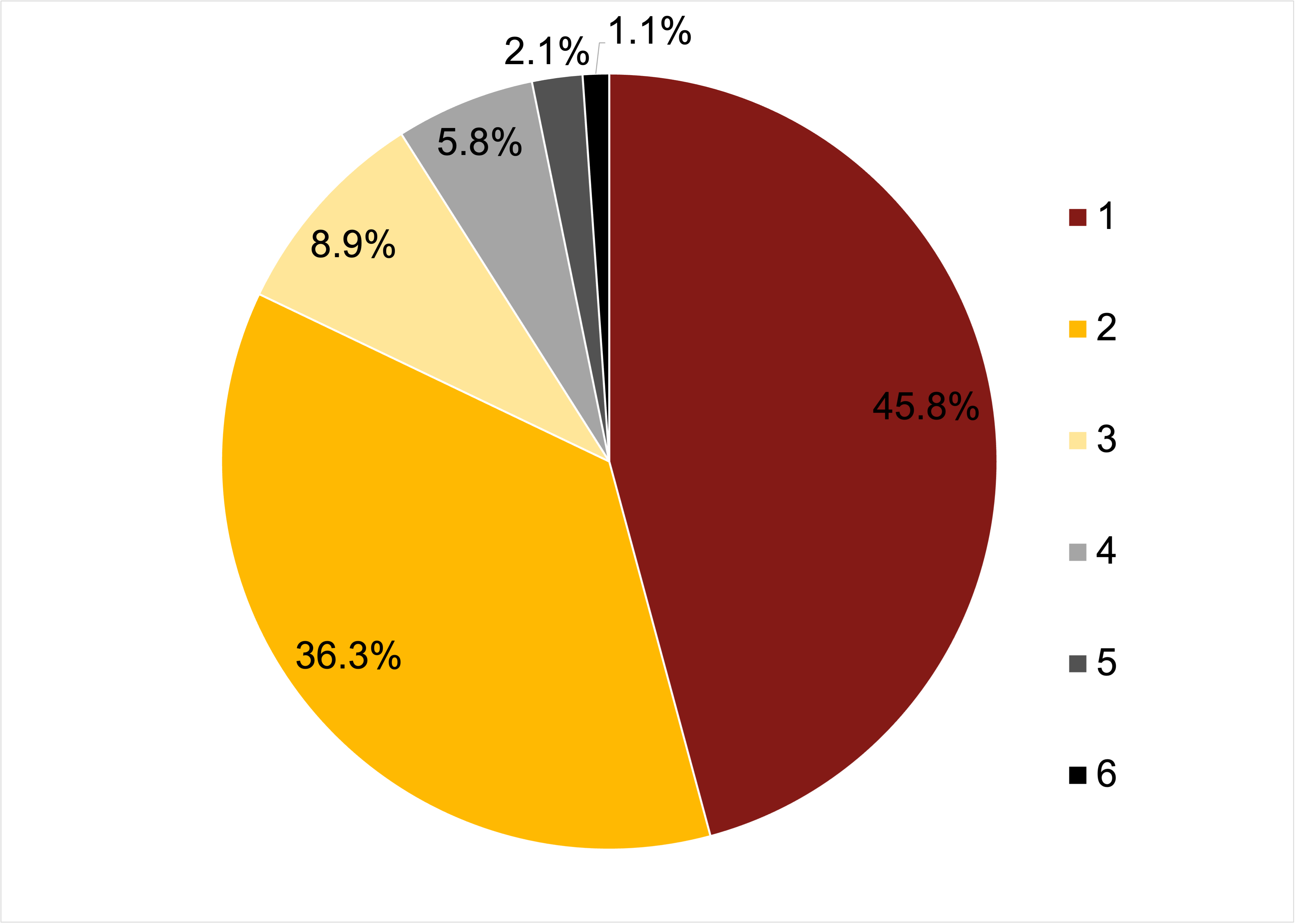
**

**Supplemental Figure 2.** Empiric urinary tract infection (UTI) antibiotic therapy among patients (n = 157) with a urine culture (UC) reflex and no concurrent non-UTI infections.


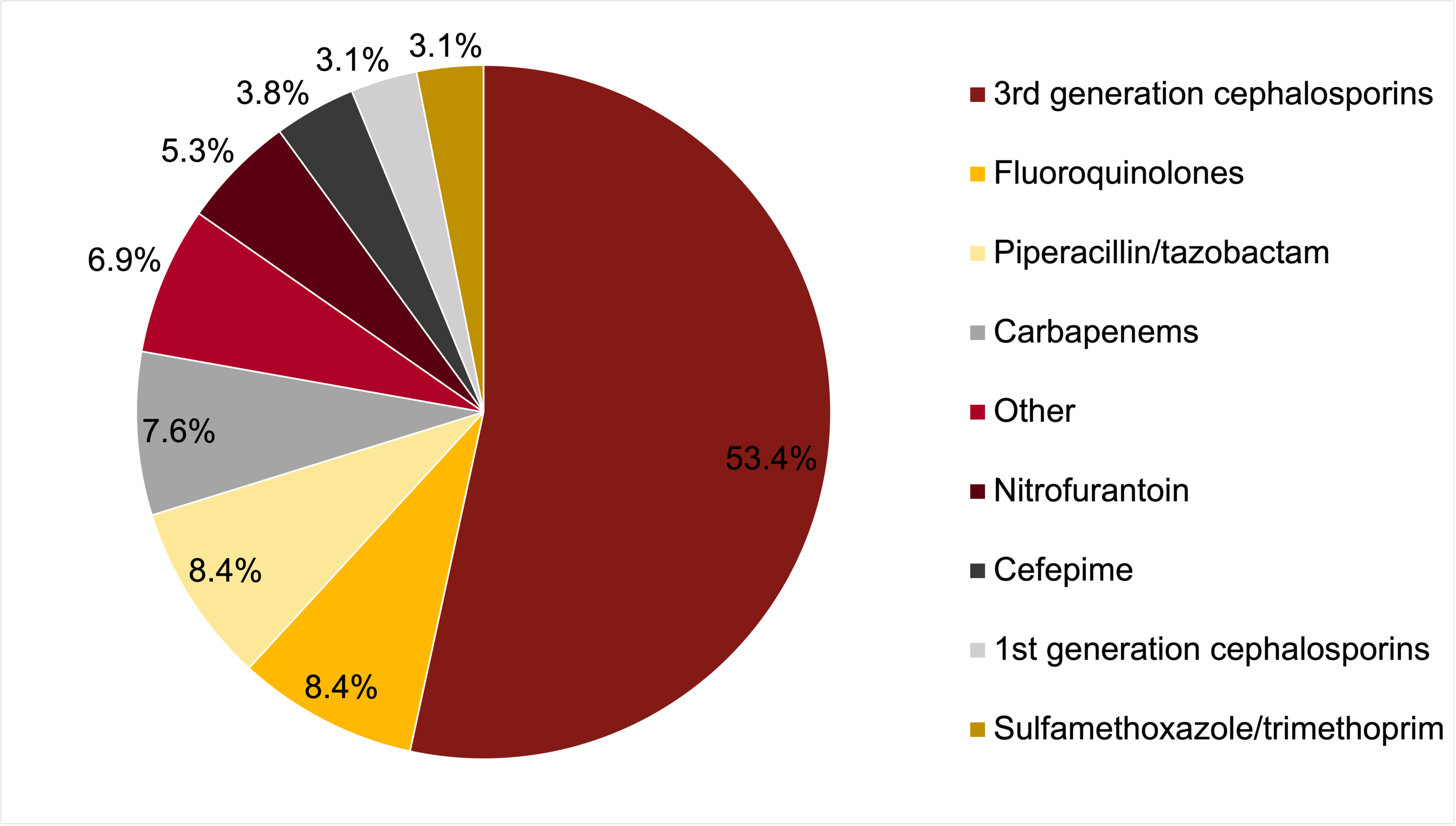


Other: vancomycin, amoxicillin/clavulanate, linezolid, ampicillin/sulbactam

**Supplemental Figure 3.** Definitive urinary tract infection (UTI) antibiotic therapy among patients (n = 157) with a urine culture (UC) reflex and no concurrent non-UTI infections.


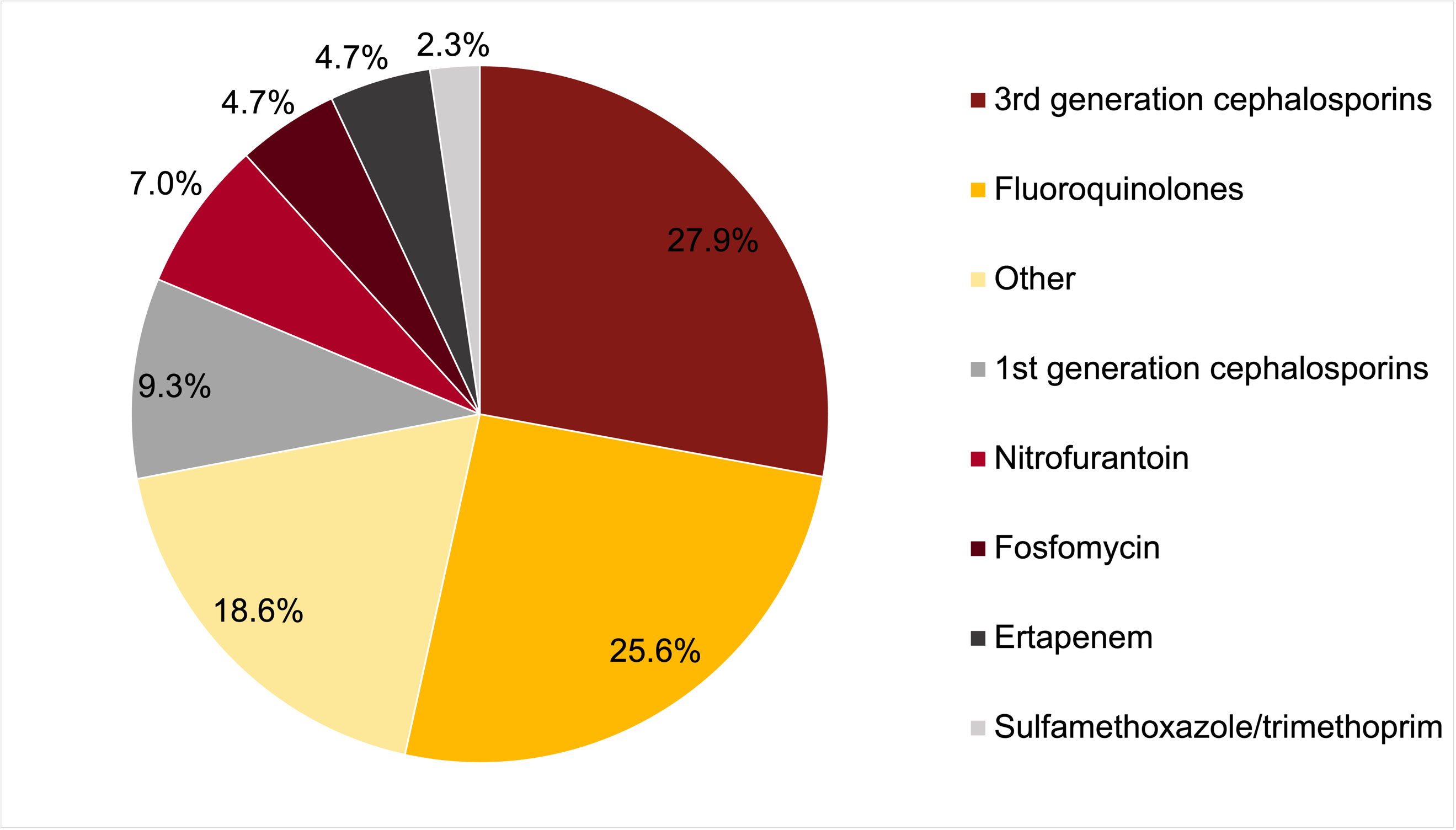


Other: amoxicillin, amoxicillin/clavulanate, ampicillin, ampicillin/sulbactam, vancomycin
